# 

**Published:** 1950-10

**Authors:** 


### CONTENTS

| | Page |
|---|---|
| **The Prognosis in Hypertension.** By D. H. DAVIES, M.D., M.R.C.P. | 103 |
| **The Surgical Treatment of Hypertension—I.** By G. L. ALEXANDER, F.R.C.S. | 110 |
| **The Surgical Treatment of Hypertension—II.** By ASHTON MILLER, F.R.C.S. | 115 |
| **Editorial Note** | 120 |
| **Reviews of Books** | 120 |
| **Meetings of Societies** | 124 |
| **Local Medical Notes** | 126 |

